# Solvents and Stabilization in Ionic Liquid Films

**DOI:** 10.1021/acs.langmuir.2c01258

**Published:** 2022-07-21

**Authors:** Andrew Horvath, Radhika S. Anaredy, Scott K. Shaw

**Affiliations:** Department of Chemistry, University of Iowa, Iowa City, Iowa 52242, United States

## Abstract

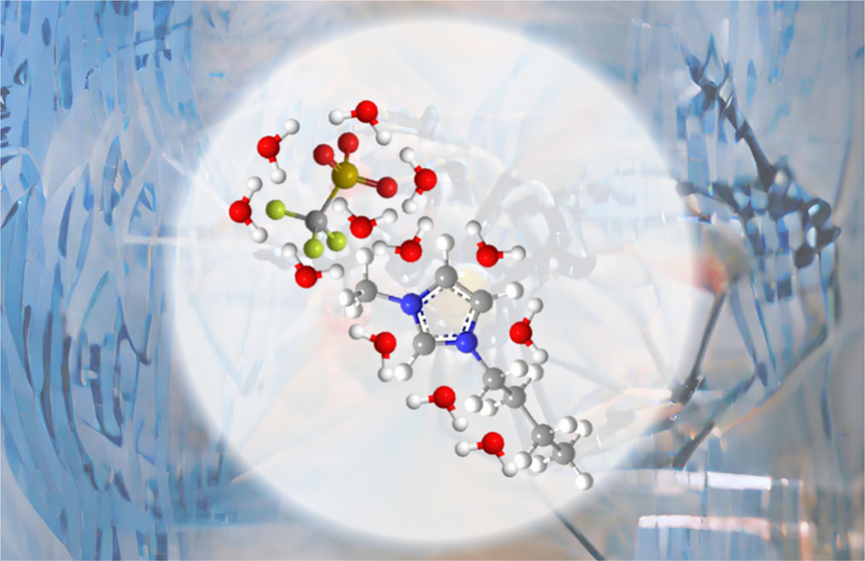

We report the interfacial structures and chemical environments of ionic liquid films as a function of dilution with molecular solvents and over a range of film thicknesses (a few micrometers). Data from spectroscopic ellipsometry and infrared spectroscopy measurements show differences between films comprised of neat ionic liquids, as well as films comprised of ionic liquids diluted with two molecular solvents (water and acetonitrile). While the water-diluted IL films follow thickness trends predicted by the Landau–Levich model, neat IL and IL/MeCN films deviate significantly from predicted behaviors. Specifically, these film thicknesses are far greater than the predicted values, suggesting enhanced intermolecular interactions or other non-Newtonian behaviors not captured by the theory. We correlate film thicknesses with trends in the infrared intensity profiles across film thicknesses and IL-solvent dilution conditions and interpret the changes from expected behaviors as varying amounts of the film volume existing in isotropic (bulk) vs anisotropic (interfacial) states. The hydrogen bonding network of water-diluted ionic liquids is implicated in the agreement of this system with the Landau–Levich model’s thickness predictions.

## Introduction

Chemical interfaces are crucial components of various biological and physical systems. Previous studies to understand interfacial structures have taken many approaches, including spectroscopic and mechanical probing, as well as density functional theory (DFT) and molecular dynamics (MD) simulations. Results from these studies commonly show nanoscale domains that form spontaneously between two phases of matter to form the chemical interface. Importantly, these microscopic volumes of matter have fundamentally different physical properties and behaviors than those of the same materials found in the adjacent bulk phases. These properties can affect many (bio)chemical processes, so understanding the fundamental interactions and ramifications is important. The molecular organizations and distal extents of interfacial domains are often linked to shapes, sizes, and intermolecular interactions of the species present.^1−11^ As the molecules at the interface become more complex, the available interactions generally increase. This makes ionic liquids (ILs) particularly interesting because they are composed entirely of intentionally bulky and asymmetric molecular ions that encompass a wide range of intermolecular interactions.^12−15^ The steric bulk works to frustrate crystallization allowing ILs to remain liquid at or even below room temperature and creates a rich diversity of interfacial molecular architectures, sometimes extending beyond the few nanometers of the traditionally defined chemical interface.^16,17^ For instance, Atkin and Israelachvili et al. showed ordering in IL systems up to 10 nm from the surface by the use of atomic force microscopy, while Swain and Blanchard et al. used confocal fluorescence anisotropy to demonstrate free charge density gradients that can range ca. 100 μm from a solid surface.^7,18,19^ This is in contrast to other work showing the ordered region extends to less than 1 nm from a solid surface.^20−23^ In general, it has also been reported that liquids become increasingly solid-like as they are confined between two surfaces.^24^ Espinosa-Marzal et al. used a surface force apparatus to study an IL film under nanoconfinement between two parallel interfaces as well as the effects of shearing when these interfaces are slid past one another. They found that under nanoscale confinement, the IL fluid adopts a solid-like structure resulting in large changes to physical properties including 100-fold increases in viscosity.^25^

Ionic liquids are generally hygroscopic, and many can be readily dissolved in water or other molecular solvents, e.g., acetonitrile (MeCN).^26,27^ Water is ubiquitous and a popular cosolvent for IL systems,^28^ and it can often be considered an “impurity” in ILs due to their hygroscopic nature. Most protic ILs are completely miscible with water, while some aprotic ILs exhibit upper limits of water miscibility, after which water may be still absorbed in the liquid but often forms aqueous-rich domains, or nano-droplets, yielding a heterogeneous mixture.^29−33^ The presence of water or other cosolvents influences ILs’ bulk and interfacial properties. An extensive study on molecular states of water in bulk imidazolium-based ILs with different anions has been carried out by Welton et al. using Fourier transform infrared (FTIR) and attenuated total reflection (ATR). The results of this study displayed that higher basicity of the anions leads to a stronger interaction with water molecules as indicated by a diagnostic shift in the O–H stretching absorption profile.^34^

Brennecke et al. reported varying degrees of IL–water interaction across selected compositions of cations and anions based on measurements of excess enthalpies for these mixtures.^35^ A study on interfacial water–IL systems reported by Baldelli et al. shows that while water tends to interact strongly with ILs in water-miscible ILs, in specifically water-*immiscible* ILs, water does solvate IL cations at the interface as evidenced from orientational changes in cation dipoles.^36^ Conboy et al. also studied interfacial water structure at water/SiO_2_ and RTIL/SiO_2_ interfaces using SFG. They reported identical ice-like and water-like features at both interfaces. At the RTIL/SiO_2_ interface, water was found to hydrogen bond with imide-based ILs to form monomeric or dimeric complexes (a water molecule bridging two anions). The concentration of singly versus doubly coordinated water molecules was determined from the intensity of free O–H resonance at 3700 cm^–1^ versus the hydrogen-bonded O–H–N peak at 3500 cm^–1^. At lower water content, a water molecule bridges between two anions forming a dimer. At higher water concentrations, the free O–H intensity increases as more water molecules are hydrogen bonded to a single anion. Espinosa-Marzal et al. studied the effects of water absorption on the structure and dynamics of hydrophilic and hydrophobic ILs confined between hydrophilic mica surfaces at a distance of ∼10 nm using surface force apparatus. It was found that with hydrophobic IL at RH above ca. 45% (ca. 9–1 IL/water mole ratio), surface-induced phase separation occurs due to immiscibility of IL with water and stronger interaction of water with mica and eventually water replaces the IL in the nanopores.^25^ In the case of hydrophilic ILs (ca. 2.4–1 water/IL mole ratio), water intercalates in the IL affecting the molecular packing and density but did not affect layering near solid surfaces, and the viscosity of “wet” IL was found to be an order of magnitude lower than the corresponding dry IL.^25^ ILs also have a wide electrochemical stability window and high charge density compared to other solvents, which make them attractive as (capacitor) electrolytes, but high viscosities for the neat liquids reduce their conductance.^37^ Viscosity increases further in confined interfacial regions, which plays an important role in many (electrochemical) processes.^25^ Hence, it is desirable to understand the physicochemical properties of ILs diluted with suitable cosolvents.

Endres et al. carried out spectroscopic and AFM studies on 10 to 70 vol % water in 1-ethyl-3-methylimidazolium trifluoromethylsulfonate ([Emim][OTf]) ionic liquid. The results of the AFM studies show that the addition of water alters the interfacial region drastically with the interaction of cation and anion completely destroyed above 50 vol % water where both ions are completely solvated by water molecules. Neat IL solvents form multilayers at a solid surface and hence do not follow Gouy–Chapman–Stern theory, but even small amounts of water (<30% vol) can alter the interface to form double-layer-like aqueous electrolytes, which significantly alters interfacial processes.^21^

Recent work by Aparicio et al. showed that organic solvents, e.g., acetone and MeCN, show a strong affinity for the hydrogen bond donor sites on the imidazolium cation in apolar–aprotic ILs ([BMIM][BF_4_] and [BMIM][PF_6_]). This led to a disruption of the bulk ionic liquid structure as the organic solvents were competing with the anions to pair with the imidazolium cations.^27^ These results are comparable to yet distinct from results acquired by Yu et al. who found that acetonitrile causes clusters of [BMIM][BF_4_] ions to break apart into discrete ion pairs surrounded by MeCN molecules but the MeCN was incapable of overcoming the relatively strong Coulombic interactions between anion–cation pairs.^38^

Given the utility of ionic liquids, and their surprisingly different and often desirable behaviors in the presence of cosolvents, our group probes the IL interface to determine the IL behaviors. To effectively probe these domains, we pair spectroscopic analysis with a novel dynamic wetting technique to compare the effects of water (protic) and acetonitrile (aprotic) cosolvents mixing with ionic liquids 1-butyl-3-methylimidazolium triflate [BMIM][OTf] and diethylmethylammonium triflate [N221H][OTf]. We hypothesize that the water/IL films will show fundamentally different spectral features owing to water’s ability to form strong hydrogen bonds. This will most likely lead to a more organized and more stable interfacial region within the film. We do not expect the entirety of the film to be organized, but rather as in previous work, we expect to see an organized interfacial domain as well as a disordered “bulk” domain.^39^ The two fluid mixtures are prepared to exhibit nearly identical bulk properties, and we report their behaviors in films ranging from 10 to 1000 s of nanometers of thickness, spanning the “bulk” to “interfacial” transition.

## Experimental Section

### Materials

Spectroscopic measurements are acquired in a reflection-based geometry using 14 mm diameter polycrystalline silver disks as solid, reflective substrates. Ag disks are cut from a 99.999% purity silver rod (ESPI metals, Portland, OR) and polished to a mirror finish using progressively finer grit polishing media, starting with 600 then 1000 grit sandpapers and followed by 9.5, 3.0, 1.0, and 0.3 μm aluminum oxide powder on Buehler polishing pads. The surfaces are finally polished chemically using a chromic acid etch to reveal a clean, smooth, and reflective surface.^40^ Ag surface roughness and optical constants are measured by atomic force microscopy (AFM) and spectroscopic ellipsometry, respectively. The RMS roughness is ≤3 nm and the *n* and *k* values determined by ellipsometry match those reported for the clean, bare metal.^41^

The ionic liquids 1-butyl-3-methylimidazolium triflate [BMIM][OTf] (99%, Iolitec, Heilbronn, DE) and diethylmethylammonium triflate [N221H][OTf] (98%, Iolitec, Heilbronn, DE) are purchased and dried under reduced pressure for > 5 days to remove residual water and volatile impurities. All ionic liquids are stored in a glovebox under a nitrogen atmosphere when not in use. Water content is monitored before each experiment via Karl Fischer titration (see below). Ultrapure water used for dilutions here is generated by a Milli-Q UV Plus System (Millipore Corp) and is consistently at 18.2 MΩ cm^–1^ with TOC ≤4 ppb. Acetonitrile (MeCN) (99% Millipore Corp) is dried and deoxygenated using a solvent purification system (Pure Process Technologies, Nashua NH) and stored over 3 Å molecular sieves under a nitrogen atmosphere until used.

### Instrumental Methods

#### Karl Fischer

Water content is determined using a Metrohm 831 Karl Fischer (KF) titrator with a two-reagent diaphragm cell. The outer cell consisted of a Hydranal Coulomat AG anolyte (Fluka Analytical) or Aqualine Electrolyte AG (Fisher Chemical) and the inner cell of a Hydranal Coulomat CG catholyte (Fluka Analytical). Hydranal Water Standard 1.0 (Fluka Analytical) is used to calibrate the instrument after changing the Karl Fischer reagents and periodically during these experiments. The sample is stirred vigorously before every Karl Fischer measurement to ensure homogeneity. IL sample sizes of ca. ∼200 μL (0.25 g) are used for the KF measurements and every measurement is replicated at least in triplicate.

#### Contact Angle

Surface tension measurements are made using a contact angle goniometer (Rame-Hart model 100) modified in-house to utilize a 1280 × 1024-pixel monochrome CMOS camera and a 6-60X magnification objective (Thor Labs). Drop shape analysis is performed using ImageJ software to determine the surface tension of each solution.

#### Viscosity Measurements

Viscosity measurements are performed using a Brookfield model DV2T-LV rotational viscometer in the cone and plate geometry. Measurements are acquired at 25 °C. Constant temperature was achieved via a temperature controlling circulating bath (Brookfield model TC-550), which recirculates a glycol/water solution through a jacketed sample cup on the viscometer.

#### Dynamic Wetting

Wetting experiments are performed using an air-tight PTFE cell and motor assembly described previously.^42,43^ Briefly, the disk-shaped metallic substrates are rotated at varying velocities through a fluid droplet dispensed by a glass capillary. This causes a fluid film to be extruded onto the reflective substrate. This film is probed with various spectroscopic techniques. The interior of the cell is purged with dry N_2_ gas or other gases and vapors as desired to control for exposure to water, oxygen, etc. In these measurements, the cell is saturated with the corresponding solvent vapors to control evaporation. The films created in this study are all more than 100 nm thick, which is visible to the eye. Visual monitoring combined with consistent MSE values from ellipsometry measurements confirm that the fluid films remain as films and do not rupture into droplets during the course of these measurements.

#### FTIR

A Thermo-Nicolet iS50 Fourier transform spectrometer with liquid N_2_ cooled MCT-A detector is used to acquire FTIR spectra. For transmission measurements, a ca. 10 μL aliquot of the sample is pressed between two CaF_2_ plates, and spectra are averaged over 128 scans at 4 cm^–1^ resolution. Duplicate spectra are obtained for each sample. The CaF_2_ plates are cleaned using copious ultrapure acetone rinses followed by drying under a dry nitrogen stream in between measurements.

#### IRRAS

Spectroscopic characterization of the films is performed in a reflection geometry using infrared reflection absorption spectroscopy (IRRAS). IRRAS spectra are acquired using a Thermo-Nicolet iS50 FTIR spectrometer coupled to an external optical bench built in-house to accommodate the dynamic wetting cell. The external bench passes the infrared light through a wire grid polarizer to create a p-polarized incident beam. This beam is gently focused onto the sample at an incident angle of 78 ± 3° from normal. All spectra are collected at 4 cm^–1^ resolution and averaged over 1000 scans. Background spectra are collected from clean and dry substrates before introducing the fluid film.

#### Ellipsometry

An M-2000 spectroscopic ellipsometer (J.A. Woollam Co., Inc.) is used to measure film thicknesses and optical properties. The ellipsometer reports ψ and Δ values as a function of wavelength from 350 to 1000 nm. These values contain information to calculate the refractive index, extinction coefficient, and thickness of the film as experimental conditions are varied. The fitting model for systems reported here consists of a bare silver substrate layer, an intermixed layer, and a general oscillator layer to account for some small optical absorption by the thickest films.

## Results and Discussion

The Landau–Levich model, described by eq 1, is a useful relationship of Newtonian fluid film’s thickness on a solid substrate as it is withdrawn from a fluid reservoir.^44^ As the equation shows, the film thickness (*h*) is proportional to the velocity of withdrawal (*V*) raised to 2/3 power. Film thickness is also directly proportional to the viscosity of the fluid but inversely proportional to the surface tension and density, properties that are constant at constant temperature and for a single fluid composition. In order for the Landau–Levich model to be a valid method of predicting film thickness, some key assumptions must be made. First, it is assumed that the surface area of the solid substrate (in this case, a rotating disk) is much greater than the film thickness. Second, the Landau–Levich model assumes the film will wet the surface and not form droplets. This is a consequence of the surface tension of the fluid being sufficiently low such that the interaction between the substrate and the fluid is the dominant force. Finally, the Landau–Levich model assumes that the solid substrate is moving at a sufficiently slow velocity such that the first molecular layer of fluid in contact with the substrate can be considered static with respect to the velocity of the substrate.^1^ We believe that the Landau–Levich model is valid for use with the films created in this study, for the following reasons. First, the substrates used in this study are 14 mm in diameter, which is several orders of magnitude greater than the thicknesses of the films. Second, we are able to visually confirm that the films are not dewetting from the surface. Finally, previous work from our group has shown that the substrates are sufficiently smooth to treat the boundary layer as static.^45,46^ Some ILs are non-Newtonian fluids and are not necessarily expected to follow the Landau–Levich model. Their physicochemical properties that lead to deviations from this model are fundamentally interesting.^39,42,43,47−49^ The work presented here aims to determine how mixing ILs with traditional Newtonian molecular solvents, e.g., water and acetonitrile, affects these physicochemical behaviors. We are particularly interested in the thin film structure as it includes the “interfacial” and “bulk” phases and the dynamics of the IL system converting between these two phases.
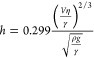
1

This is the Landau–Levich model of film thickness as a function of withdrawal velocity, where *h* = thickness (m), *V* = velocity of withdrawal (m/s), η = viscosity (Pa*s), ρ = density (kg/m^3^), *g* = acceleration of gravity (m/s^2^), and γ = surface tension (N/m).

We chose two protic ILs to study the effect of cosolvents on interfacial behavior, 1-butyl-3-methylimidazolium triflate [BMIM][OTf] and diethylmethylammonium triflate [N221H][OTf]. The chemical structures and selected physical properties for these two ILs are shown in Table 1, right column. The neat liquids have been studied previously by us and other groups.^43,47^

**Table 1:**
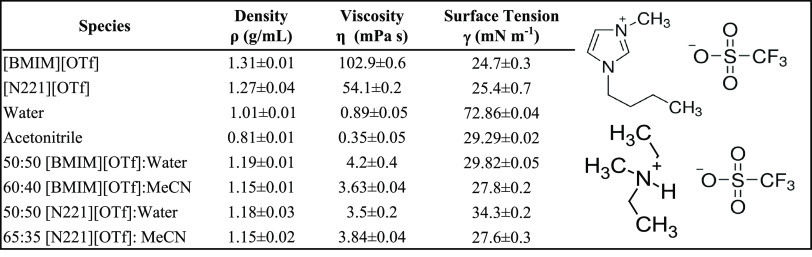
Physical Properties of the Neat IL, Solvents, and IL Mixtures Used in This Study

In this work, thin films of the IL–cosolvent mixtures supported on the Ag substrate were made using a dynamic wetting technique described above and in previous work.^43,50,51^ This technique makes use of a glass capillary to dispense a drop of the fluid mixture at the bottom of the silver substrate, which is rotated by a motor through this bulk drop, forming a thin film that is probed by the IR beam at the top of the surface. Theoretical film thicknesses, as determined by the Landau–Levich model, are dependent on the bulk properties of the fluid. IL–cosolvent mixtures are chosen to keep the viscosities, densities, and surface tensions of all fluids examined here similar to each other. This means that within the Landau–Levich model and in the absence of specific intermolecular interactions, the fluids’ thin film properties should be similar. Table 1 lists the compositions of the neat and mixed liquid solutions used in these studies. Table 1 also includes select physical properties of the neat ILs, cosolvents, and mixtures.^52,53^ Density was measured by gravimetric analysis. Viscosity was measured via a rotational viscometer. Surface tension was measured using the pendant drop method. The values we observe are either very close to known literature values or expected from known trends in the physical properties of mixtures.^54−58^

**Figure 1:**
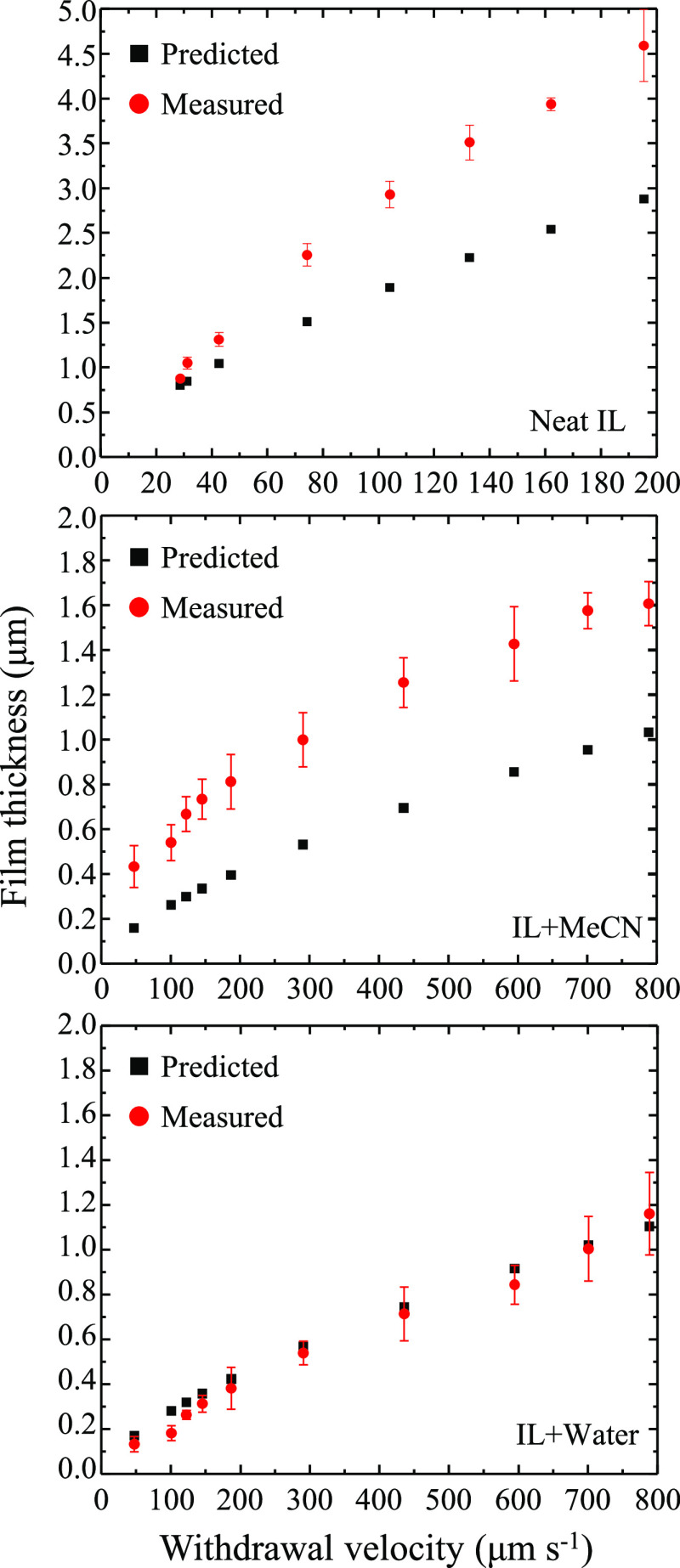
Predicted (black squares) and measured (red circles) film thicknesses measured by spectroscopic ellipsometry for [BMIM][OTf] (top), [BMIM][OTf] and acetonitrile (middle), and [BMIM][OTf] and water (bottom). Data points represent replicate measurements where *n* ≥ 3. Error bars represent standard deviations in the measurement.

The liquid mixtures in this study are designed to exhibit similar densities, viscosities, and surface tensions. This simplifies comparisons between the chemical systems and aids in developing relationships of microscopic intermolecular interactions and molecular architectures with macroscopic properties. In this vein, Figure 1 shows the film thickness of ILs and mixtures with MeCN and H_2_O as a function of rotational velocity as measured by spectroscopic ellipsometry. Numerical data tables for these data are provided in the Supporting Information (Figure S1). The data for the neat IL (top) and 60/40 [BMIM][OTf]/MeCN mixture (middle) show significant deviations from values predicted by Landau–Levich. Both of these fluids’ films display a positive deviation of experimentally measured film thicknesses when compared to the Landau–Levich model predictions. The deviation increases at higher velocities (larger film thicknesses). Figure 1 (bottom) also shows calculated and measured film thickness for 50/50 [BMIM][OTf]/water. Despite the nearly identical density, surface tension, and viscosity to the [BMIM][OTf]/MeCN mixture, the [BMIM][OTf]/water films show a very close agreement, within experimental errors, between predicted and measured thicknesses. Only two points, corresponding to thicknesses ca. 300 nm, show slight deviations from the theoretical prediction. We suggest that the differences in the water and MeCN mixtures are attributed to differences in intermolecular interactions and specifically the hydrogen bonding character of water. The following data and discussion characterize these interactions and the films’ relevant physicochemical properties.

**Figure 2:**
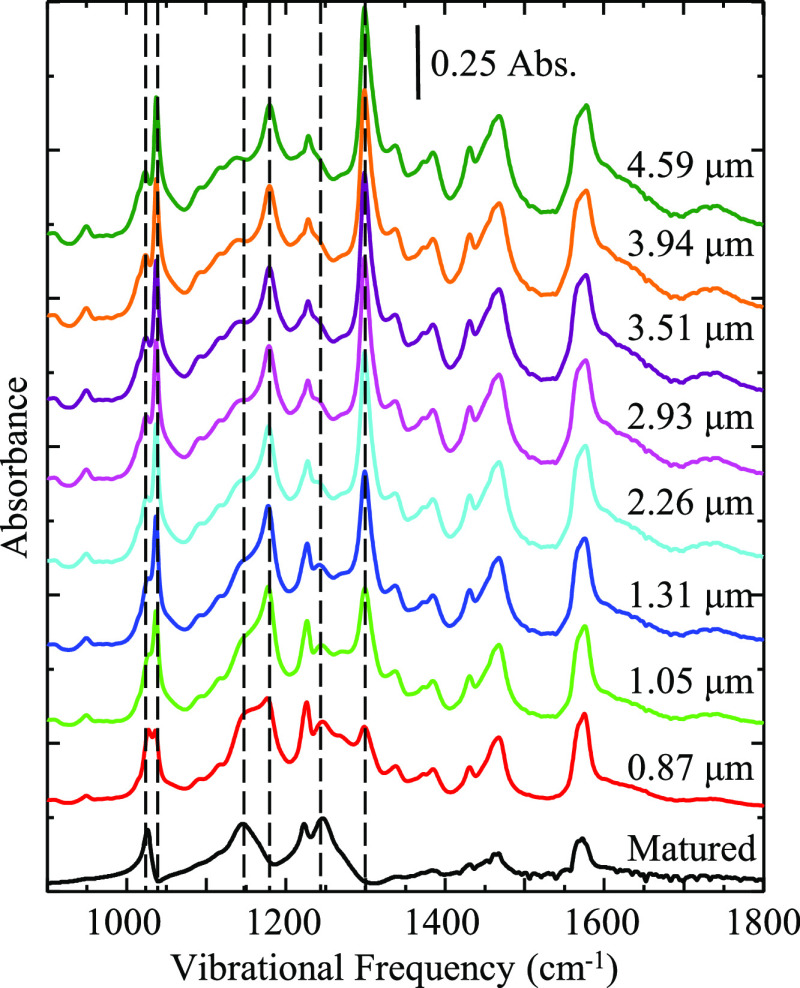
Series of IRRAS spectra showing the changing behavior of pure [BMIM][OTf] film as a function of film thickness. The bottom trace represents the matured IL film after the substrate rotation is stopped. Above this are the IR traces of the IL film acquired while the substrate is rotating. Films are prepared on a silver substrate using the dynamic wetting technique. Spectra are representative of *n* ≥ 3 trials.

Figure 2 shows a series of IRRAS spectra taken on neat [BMIM][OTf] as a function of film thickness. As substrate velocity increases the film thickness also increases, and changes in the spectral profile are indicative of new chemical environments and molecular orientations within the fluid film. Generally, the absorbance intensity increases with increasing film thickness, which is expected according to the Beer–Lambert law. What is interesting in this case is that vibrational modes assigned to an isotropic “bulk” environment increase at a faster rate with increasing substrate rotation than modes assigned to an anisotropic “interfacial” environment.^47^ Our previous work on [BMIM][OTf] and [N221H][OTf] films showed that while the film was rotating at a constant speed of 60 μm/s, it consisted of two different environments, interfacial and bulk, previously identified by two absorption peaks for each of the anion’s vibrational modes.^47^ Here, we focus on the SO_3_ symmetric stretch and CF_3_ asymmetric stretch, which are located at ca. 1030 and 1170 cm^–1^, respectively, as seen in Figure 2. In the film environments probed here, both of these modes are split into two overlapping but clearly identified absorptions, which correspond to molecules present in isotropic and anisotropic environments. For both modes, higher energy absorptions are associated with the isotropic environment relative to the lower energy band associated with the anisotropic environment. This is confirmed by comparing the thin film spectra acquired here to the “bulk” infrared spectra acquired in traditional transmission infrared measurements.^43,47^ Specifically, absorption for the SO_3_ symmetric stretch is split into two peaks at 1027 and 1038 cm^–1^, and the CF_3_ asymmetric stretch is split into absorptions at 1160 and 1180 cm^–1^. Differences in the degree of splitting are rationalized by differences in the chemical environments of these modes between the “interface” and “bulk.”^59−61^ Other significant changes include the (isotropic) SO_3_ asymmetric stretch at ca. 1300 cm^–1^ growing in relative absorbance and sharpening, while the (anisotropic) SO_3_ asymmetric stretch at 1250 cm^–1^ remains constant with respect to the changing film thickness. As the films thicken, the increase in higher energy vibrations implies that more bulk (disordered) mass is contained within the film volume.

**Figure 3:**
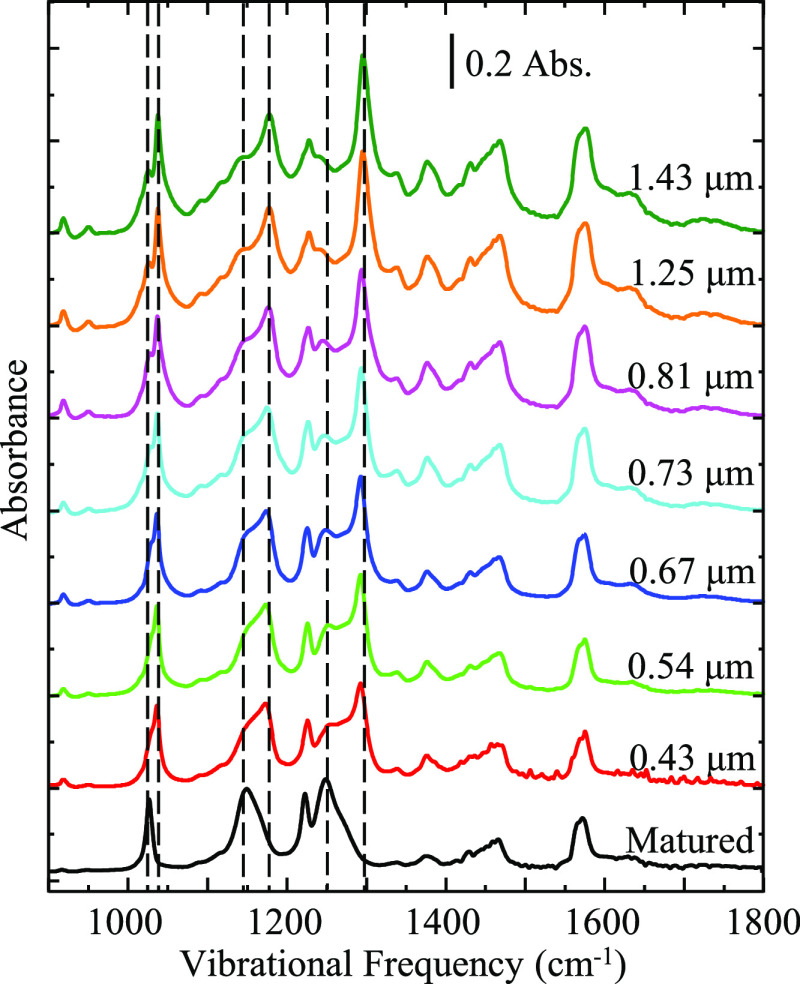
Series of IRRAS spectra showing the changing behavior of a 60/40 [BMIM][OTf]/MeCN film as a function of film thickness. The bottom trace represents the matured IL/MeCN film after the substrate rotation is stopped. Above this are the IR traces of IL/MeCN mixture acquired while the substrate is rotating. Films are prepared on a silver substrate using the dynamic wetting technique. Spectra are representative of *n* ≥ 3 trials.

To contrast the data for the neat IL films presented in Figure 2, Figure 3 shows a series of IRRAS spectra for mixed films comprised of 60% [BMIM][OTf] and 40% MeCN vol/vol. This corresponds to a ca. 3:1 mole ratio MeCN/IL. Like Figure 2, these data are collected and reported vs film thickness values measured by spectroscopic ellipsometry, as listed in the figure. The bottom spectrum listed as “matured” represents the infrared absorption profile of the IL/MeCN mixed film ca. 10 min after rotation of the substrate is stopped. Similar spectral changes can be seen in the mixture spectra when compared to the neat IL spectra seen in Figure 2. Specifically, the same changes seen in the neat IL films at 1027, 1038, 1160, 1180, 1250, and 1300 cm^–1^ are also present in the MeCN diluted film. Considering these similarities, it is apparent that both the neat IL film and the mixed IL/MeCN film display similar spectral profiles and behaviors. This implies that the IL:MeCN film is generally exhibiting similar intermolecular interactions and chemical environments as seen in the neat IL film. Further, there is a general increase in the absorbance of the isotropic modes relative to the absorbance of the anisotropic modes as film thickness increases. This indicates more bulk contribution to the overall spectral profile with increasing film thickness. We note that the isotropic modes become dominant in comparison to the anisotropic modes immediately upon rotation of the substrate. A similar plot of infrared spectra corresponding to a 65% vol IL mixture of [N221H][OTf] and MeCN can be found in the Supporting Information (Figure S2). Similar changes analogous to those observed in the [BMIM][OTf]/MeCN films are also observed in films comprised of [N221H][OTf]/MeCN. Based on these observations, we suggest that while MeCN is highly soluble in the ionic liquids, it does not have significant intermolecular interaction with the IL molecules and is not significantly perturbing the intermolecular architecture(s) present in the films. This is in direct contrast to the behavior observed in the IL/water films discussed below.

**Figure 4:**
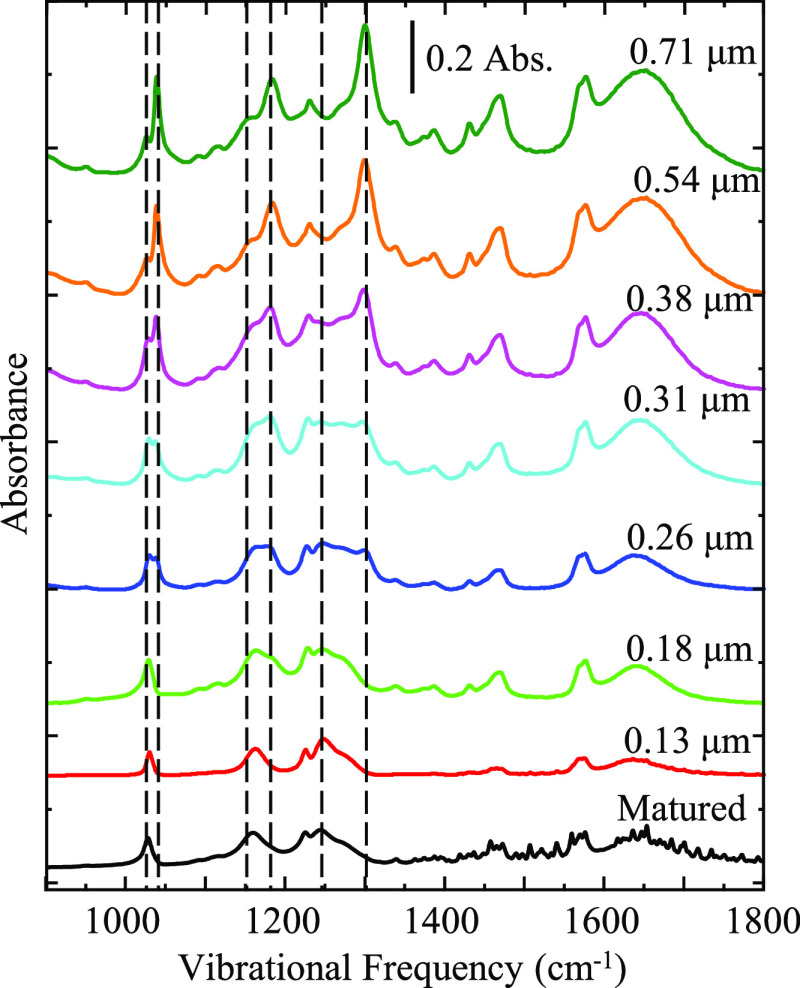
Series of IRRAS spectra showing the changing behavior of a 50/50 [BMIM][OTf]/water film as a function of film thickness. The bottom trace represents the matured IL/water film after the substrate rotation is stopped. Above this are the IR traces of IL/water mixture acquired while the substrate is rotating. Films are prepared on a silver substrate using the dynamic wetting technique. Spectra are representative of *n* ≥ 3 trials.

**Figure 5:**
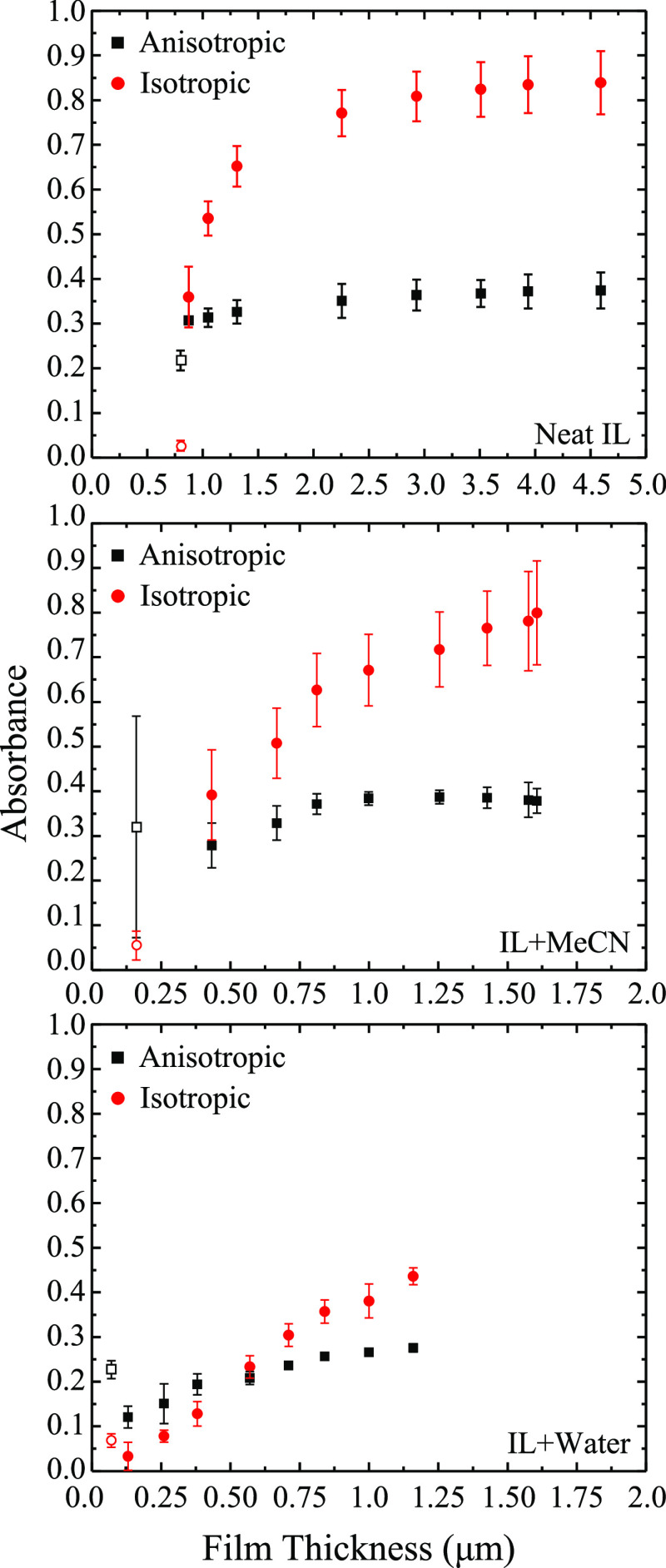
Plots identifying SO_3_ asymmetric stretch absorbance in the isotropic (1300 cm^–1^) and anisotropic (1250 cm^–1^) environments within the liquid film. Data are plotted as a function of film thickness as taken from IRRAS spectra acquired on [BMIM][OTf] (top), solutions of [BMIM][OTf] and acetonitrile (middle), and [BMIM][OTf] and water (bottom). Films are prepared on a silver substrate using the dynamic wetting technique. Open symbols represent data acquired on the matured film, while filled symbols represent data acquired on the rotating film. Data points are representative of *n* ≥ 3 trials. Error bars represent the standard deviation in the measurement.

Figure 4 shows a series of IRRAS spectra of an IL film comprised of a 50% vol IL mixture of [BMIM][OTf] and water (ca. 12:1 mole ratio water/IL) as a function of film thickness with thickness increasing from bottom to top. In contrast, in both the neat IL and IL/MeCN films wherein the spectral profile immediately switches from one dominated by anisotropic modes to one dominated by isotropic modes, the IL/water film maintains its anisotropic character at smaller film thicknesses. In other words, the peaks associated with anisotropic modes remain dominant relative to the peaks associated with isotropic modes until the film thickness increases to a critical thickness. At this point (ca. 0.38 μm > film thickness > ca. 0.26 μm), the spectral profile film shows roughly equal contributions from the anisotropic and isotropic environments. At film thicknesses > 0.50 μm, the isotropic environment begins to dominate and the spectra adopt an increasingly “bulk-like” spectral profile. This is exemplified by a shift from the matured film spectral profile to the unmatured rotating film spectral profile and is most likely due to the bulk contribution steadily increasing with thickness while the interfacial contribution plateaus. A similar plot corresponding to a 50% vol IL mixture of [N221H][OTf] and water can be found in the Supporting Information (Figure S3). Similar changes, analogous to those observed in the [BMIM][OTf]/water films, are also observed in films comprised of [N221H][OTf]/water. The films’ evolution from anisotropic (interfacial) to isotropic (bulk) is tracked and made clearer in Figure 5, which is discussed below.

**Figure 6:**
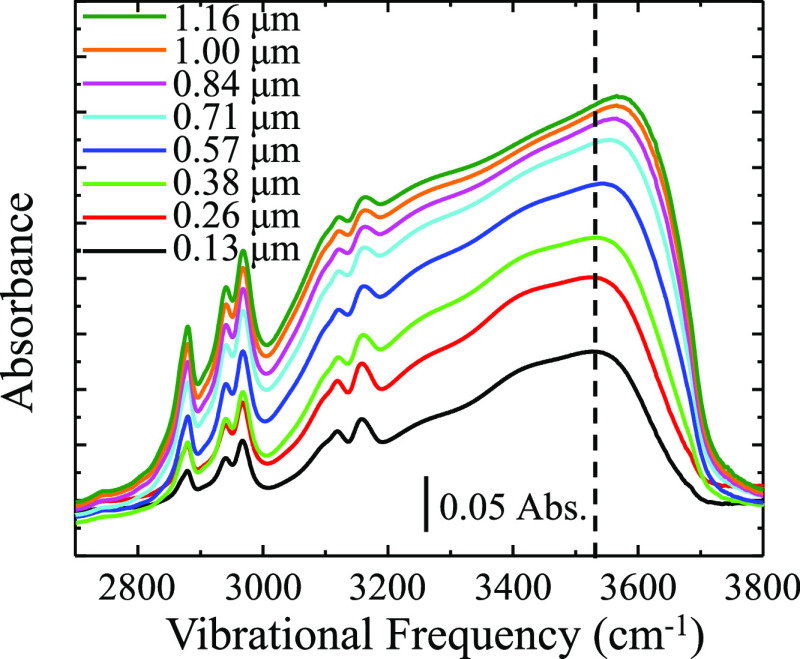
Series of IRRAS spectra showing the changing behavior in the hydrogen bonding region of a 50/50 [BMIM][OTf]/water film as a function of film thickness. The OH stretching mode shifts to a higher frequency with film thickness. Films are prepared on a silver substrate using the dynamic wetting technique. Spectra are representative of *n* ≥ 3 trials.

Figure 5 displays a plot of absorbance for the SO_3_ asymmetric stretch intensity for both isotropic (ca. 1300 cm^–1^) and anisotropic (ca. 1250 cm^–1^) environments as a function of film thickness. These data are tracked for neat [BMIM][OTf] (top), [BMIM][OTf]/MeCN (middle), and [BMIM][OTf]/water (bottom). The SO_3_ asymmetric stretch was chosen because it clearly shows relative changes in peak absorbance between the interfacial and bulk modes. Other vibrational modes discussed above display similar behavior, and plots corresponding to these changes (e.g., SO_3_ symmetric stretch and CF_3_ asymmetric stretch) are provided in Supporting Information Figures S4 and S5, respectively. Tabulated values for these data are also provided in the Supporting Information in Figures S6–S8. In all three cases, the isotropic and anisotropic peaks increase in absorbance, which is in keeping with the increased path length caused by the increasing thickness of the film per Beer’s law. Also in all three cases, the thinnest films have dominant anisotropic modes corresponding to the matured film environment as expected based on previous work demonstrating the ordered character of matured, neat IL films.^43^ There are, however, some notable differences among the three solutions. In the neat IL and IL/MeCN films, the vibrational mode associated with the isotropic environment increases in absorbance to become the dominant peak immediately upon substrate rotation. This is in direct contrast to the IL/water film wherein the vibrational mode corresponding to the anisotropic environment remains dominant even after substrate rotation resumes. Only beyond a film thickness of ca. > 0.6 μm is the shift in the spectral profile observed, which represents the isotropic environment becoming dominant. This is also apparent in Figure S9, which plots a ratio of the isotropic peak intensity to the anisotropic peak intensity of the SO_3_ asymmetric stretch for all three films (neat IL, IL/MeCN, and IL/water). In cases where this ratio is less than 1, the anisotropic environment is dominant in the film, whereas in cases where the ratio is greater than 1, the isotropic environment is dominant. It is also important to note that, in the case of the IL/water film, beyond the transition thickness of ca. > 0.6 μm, the bulk contribution steadily increases with the thickness but the contribution from the interfacial layer plateaus. This implies that up to the transition thickness, the interfacial layer also grows in the case of IL/water films. It can be plainly seen that the neat IL and IL/MeCN films immediately adopt an isotropic configuration upon substrate rotation, but the IL/water film remains in the anisotropic configuration up to ca. 0.6 μm. Ratios of the isotropic and anisotropic peaks for all modes of interest can be found in the Supporting Information (Figures S10–S12). In the cases of all three films, no significant changes are observed in the cation peaks of the spectra, which is consistent with other previous works of this type.^43^ This is shown in Figure 6, which is a plot of the high-frequency region (2700–3800 cm^–1^) of vibrational spectra acquired on a [BMIM][OTf]/water film.

**Figure 7:**
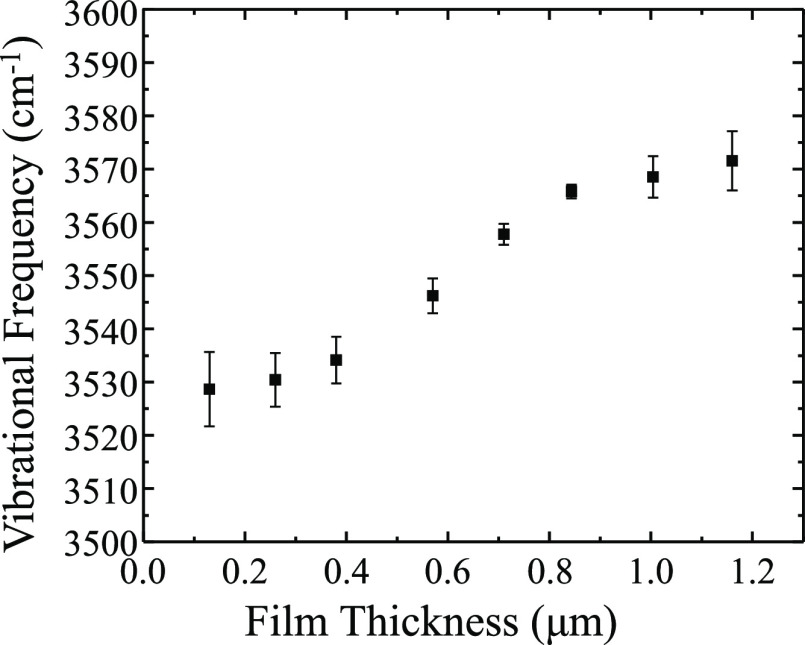
Plot showing the shift in the center of frequency on the OH stretching peak in a 50/50 [BMIM][OTf]/water film as a function of film thickness. Data are taken from IRRAS spectra shown in Figure 7. The OH stretching mode shift suggests less hydrogen bonding present at higher film thickness. Films are prepared on a silver substrate using the dynamic wetting technique. Spectra are representative of *n* ≥ 3 trials. Error bars represent the standard deviation in the measurement.

Figure 6 displays the aliphatic and OH stretching regions of the IL/water film as a function of film thickness. The broad OH stretch envelope adds intensity and shifts to a higher frequency with increasing film thicknesses, while the frequency of the aliphatic modes remains constant. The frequency shift of the OH stretch can be more clearly seen in Figure 7, which is a plot of the peak frequency of the OH stretch as a function of film thickness. There is a vast literature on the hydrogen bonding environments of water, and this blue shift of the OH bonding envelope by ca. 70 cm^–1^ for increasing film thicknesses indicates a change in the hydrogen bonding to an increasingly liquid-like state, with fewer hydrogen bonds than are present in the thinner, more ordered film environments.^31,62−64^ Similar changes are present in the [N221H][OTf]/water film and can be seen in Figure S13 in the Supporting Information.

These results suggest that the IL/water films are behaving in a Newtonian manner which, despite the similar bulk viscosities, contrasts with the IL/MeCN diluted films, which remain non-Newtonian.^48,49^ Our results imply that the water’s ability to hydrogen bond is stabilizing the relatively ordered (anisotropic) water-IL films even when the substrate is rotating at lower velocities. This is different from what we have previously reported for neat IL systems wherein extended interfacial structure is only observed in mature films and dissipates when rotation is resumed. The IL/MeCN mixed films, like the neat IL films, also immediately return to their isotropic state on resuming substrate rotation.

Previous work by Voth et al. has reported ordered microstructures in bulk ILs, supported by hydrogen bonding between polar IL head groups and water. They suggested that IL microstructures were most ordered when the available hydrogen bonds of IL are saturated with available water molecules.^65^ This occurs between a 2:1 to 3:1 water to IL mol ratio (ca. 70–80% mol fraction or 15–20% vol water). When the water content meets or exceeds this level, the hydrogen bonding network of water becomes the dominant structure in the liquid. A 50 vol % IL:water film, such as we have studied here, corresponds to a ca. 12:1 mol ratio of water:IL.^30,66^ As seen in Figures 6 and 7, there is a minimal change in the OH stretching frequency at ca. 3530 cm^–1^ up to a film thickness of ca. 0.38 μm, followed by a significant shift to higher vibrational energy from film thicknesses of 0.57–0.84 μm. Beyond thicknesses of 0.84 μm, the OH stretching frequency stabilizes at ca. 3570 cm^–1^. This shift to higher energy suggests that water’s hydrogen bonding environment shifts to weaker hydrogen bonding with itself in the thicker films.^67^ This implies that in sufficiently thick films, water forms ice-like hydrogen bonding structure even above the critical (70–80% mol fraction) amount of water content for supporting microstructures within the bulk IL suggested by Voth et al. This could be due to microscopic segregation of water into droplets in the thinner, ordered films. While there are differences between IL systems studied here and solutions of inorganic salts in water, the results are in agreement with previous studies by the Shultz and Allen groups, which demonstrate the ability of salts to disrupt the OH hydrogen bonding network.^68,69^ Furthermore, this agrees with several studies showing a low-frequency OH stretch being associated with strongly hydrogen-bonded water, while more weakly hydrogen-bonded water results in a higher energy OH stretch frequency.^31,51,70−76^ Confirming the possible segregation of water within these films is ultimately beyond the capabilities of the infrared techniques used in this study.

In contrast, work from Wang and co-workers suggests that MeCN, an aprotic solvent, does not have the same effect on IL microstructure.^26^ Their MD simulations suggest changes in physical properties and microstructures in bulk 1-butyl-3-methylimidazolium tetrafluoroborate ([BMIM][BF_4_]) diluted with a range of concentrations of MeCN and results show that MeCN molecules fill in the polar regions of the IL microstructure. At less than 70% mol fraction MeCN, there is no significant disruption in the overall IL microstructure and only a slight disruption in microstructure at 80% mol fraction MeCN.^26^ The IL:MeCN films used in our study are ca. 75% mol fraction MeCN, which suggests that the overall microstructure of the IL:MeCN systems studied here should be very similar to MD simulations conducted by Wang. Their results suggest that only slight disruption at similar MeCN concentrations is consistent with our findings of non-Newtonian behavior for both the neat IL and MeCN diluted IL films, manifested as deviations from the Landau–Levich predicted thicknesses as seen in Figure 1. This also is in general agreement with Aparicio^27^ et al. and Yu^38^ as mentioned in the Introduction section. Their combined results demonstrate the ability of MeCN to isolate ion pairs of [BMIM][BF_4_] and [BMIM][PF_6_] while avoiding any aggregation of solvent. The ionic liquid structure was significantly altered due to the inclusion of organic solvents but Coulombic interactions of anion–cation pairs were strong enough to prevent full solvation of the ionic liquid ions.

## Conclusions

To investigate interfacial structures of ionic liquids and their mixtures with water and acetonitrile, three different liquid films were examined via vibrational spectroscopy. We found that all three films adopt an ordered anisotropic spectral profile and that, despite similar bulk physical properties, the films’ thicknesses vary significantly from classical model predictions. Comparing neat IL films to those made from IL diluted by water or acetonitrile, our results suggest that the IL/water mixture displays increasingly ordered (anisotropic) structures when compared to the neat IL and IL/MeCN mixtures. This is true even when the film thicknesses increase. The transition from interfacial dominant to bulk dominant character within the film is characterized by infrared spectroscopy: in the case of the IL/water mixture films, the transition occurs only beyond a critical film thickness. These results suggest that the hydrogen bonding associated with water in the IL acts to stabilize, or facilitate the formation of, ordered structures within the IL/water films even while the substrate is rotating. This helps to explain why the films diluted with water behave consistently with the Landau–Levich model, while films diluted with MeCN do not. These results are supported by vibrational spectroscopy of the films, which shows a clear transition from ice-like OH stretching to water-like OH stretch with increasing film thicknesses. These results add significant information to the understanding of the interfacial interactions and dynamics of diluted IL systems.

## Supporting Information Available

Tabulated film thickness data from ellipsometry measurements (Figure S1); series of IRRAS spectra acquired on [N221H][OTf] diluted with MeCN and water (Figures S2 and S3, respectively); >plots of the absorbance of the SO_3_ symmetric stretch and CF_3_ asymmetric stretch as a function of film thickness (Figures S4 and S5, respectively); tabulated absorbance data of anisotropic and isotropic vibrational modes (SO_3_ asymmetric stretch, SO_3_ symmetric stretch, and CF_3_ asymmetric stretch) as a function of film thickness in films of neat [BMIM][OTf], [BMIM][OTf]/MeCN, and [BMIM][OTf]/water (Figures S6, S7, and S8, respectively) acquired by IRRAS; ratios of the absorbance of the isotropic peak to the anisotropic peak of the SO_3_ asymmetric stretch, SO_3_ symmetric stretch, and CF_3_ asymmetric stretch as a function of film thickness acquired by IRRAS on films of neat [BMIM][OTf], [BMIM][OTf]/MeCN, and [BMIM][OTf]/water (Figure S9); ratios of the absorbance of the isotropic peak to the anisotropic peak of the SO_3_ asymmetric stretch (Figure S10), SO_3_ symmetric stretch (Figure S11), and CF_3_ asymmetric stretch (Figure S12) as a function of film thickness and solvent dilution; and IRRAS spectra of the water stretching region acquired on a 50/50 [N221H][OTf]/water film as a function of film thickness (Figure S13) (PDF)
