# Supplementary material for: Solvents and Stabilization in Ionic Liquid Films
Source: Langmuir. 2022 Jul 21;38(30):9372–81. doi: 10.1021/acs.langmuir.2c01258 (PMC10111422; doi:10.1021/acs.langmuir.2c01258)
Supplement: Supplementary file 1 — la2c01258_si_001.pdf [file la2c01258_si_001.pdf]

Supporting Information for:

Title: Solvents and Stabilization in Ionic Liquid Films

Authors: Andrew Horvath[ǂ1]; Radhika S. Anaredy[ǂ1]; Scott K. Shaw*[1]

Institutions: [1]Department of Chemistry, University of Iowa, Iowa City, IA 52242, USA

Corresponding email: scott-k-shaw@uiowa.edu

Keywords: Ionic liquids, spectroscopy, FTIR, Interfaces, molten salts, water, hydrogen bonding

ǂEqual Contribution, *Corresponding Author

Figure S1: Tabulated data of predicted and measured film thicknesses.
Figure S2: A series of IRRAS spectra acquired on a 65/35 [N221H][OTf]/MeCN film as a function of film thickness.
Figure S3: A series of IRRAS spectra acquired on a 50/50 [N221H][OTf]/Water film as a function of film thickness.
Figure S4: Plots identifying the SO_3 symmetric stretch absorbance in the isotropic (1038 cm^{-1}) and anisotropic (1027 cm^{-1}) environments within neat and mixed films.
Figure S5: Plots identifying the CF_3 asymmetric stretch absorbance in the isotropic (1180 cm^{-1}) and anisotropic (1150 cm^{-1}) environments within neat and mixed films.
Figure S6: Tabulated data of peak absorbance of the isotropic and anisotropic SO_3 symmetric stretch, SO_3 asymmetric stretch, and CF_3 asymmetric stretch for [BMIM][OTf].
Figure S7: Tabulated data of peak absorbance of the isotropic and anisotropic SO_3 symmetric stretch, SO_3 asymmetric stretch, and CF_3 asymmetric stretch for 60/40 [BMIM][OTf]/MeCN.
Figure S8: Tabulated data of peak absorbance of the isotropic and anisotropic SO_3 symmetric stretch, SO_3 asymmetric stretch, and CF_3 asymmetric stretch for 50/50 [BMIM][OTf]/water.
Figure S9: Ratios of the intensity of the peaks corresponding to isotropic modes to the intensity of peaks corresponding to anisotropic modes.
Figure S10: Ratios of the intensity of the peak corresponding to the isotropic SO_3 asymmetric stretch (1300 cm^{-1}) to the intensity of the peak corresponding to the anisotropic SO_3 asymmetric stretch (1250 cm^{-1}) as a function of film thickness.
Figure S11: Ratios of the intensity of the peak corresponding to the isotropic SO_3 symmetric stretch (1038 cm^{-1}) to the intensity of the peak corresponding to the anisotropic SO_3 symmetric stretch (1027 cm^{-1}) as a function of film thickness.
Figure S12: Ratios of the intensity of the peak corresponding to the isotropic CF_3 asymmetric stretch (1180 cm^{-1}) to the intensity of the peak corresponding to the anisotropic CF_3 asymmetric stretch (1150 cm^{-1}) as a function of film thickness.
Figure S13: A series of IRRAS spectra showing the changing behavior in the hydrogen bonding region of a 50/50 [N221H][OTf]/Water film as a function of film thickness.

Neat [BMIM][OTf]			60/40 [BMIM][OTf]/MeCN			50/50 [BMIM][OTf]/Water		
Withdrawal Rate (μms^{-1})	Predicted Film Thickness (μm)	Measured Film Thickness (μm)	Withdrawal Rate (μms^{-1})	Predicted Film Thickness (μm)	Measured Film Thickness (μm)	Withdrawal Rate (μms^{-1})	Predicted Film Thickness (μm)	Measured Film Thickness (μm)
28.6	0.79946	0.87±0.03	47.5	0.15857	0.43±0.09	47.5	0.16966	0.13±0.03
31.2	0.8472	1.05±0.07	100.8	0.26186	0.54±0.08	100.8	0.28018	0.18±0.03
42.6	1.04269	1.31±0.08	122.4	0.29805	0.67±0.08	122.4	0.3189	0.26±0.02
74.3	1.51081	2.3±0.1	145.4	0.33431	0.73±0.09	145.4	0.35769	0.31±0.03
104.1	1.89169	2.9±0.1	186.9	0.39522	0.8±0.1	186.9	0.42286	0.38±0.09
132.8	2.2251	3.5±0.2	290.6	0.53044	1.0±0.1	290.6	0.56753	0.54±0.05
162.1	2.54139	3.93±0.07	435.6	0.69475	1.3±0.1	435.6	0.74334	0.7±0.1
195.5	2.87948	4.6±0.4	594.6	0.85491	1.4±0.2	594.6	0.9147	0.84±0.09
			700.6	0.95371	1.57±0.08	700.6	1.02041	1.0±0.1
			788.5	1.0319	1.6±0.1	788.5	1.10407	1.2±0.2

Figure S1: Tabulated data of predicted and measured film thicknesses measured by spectroscopic ellipsometry for [BMIM][OTf], 60/40 [BMIM][OTf]/MeCN, and 50/50 [BMIM][OTf]/water. Data represent replicate measurements where n≥3. Error represents standard deviations in the measurement.

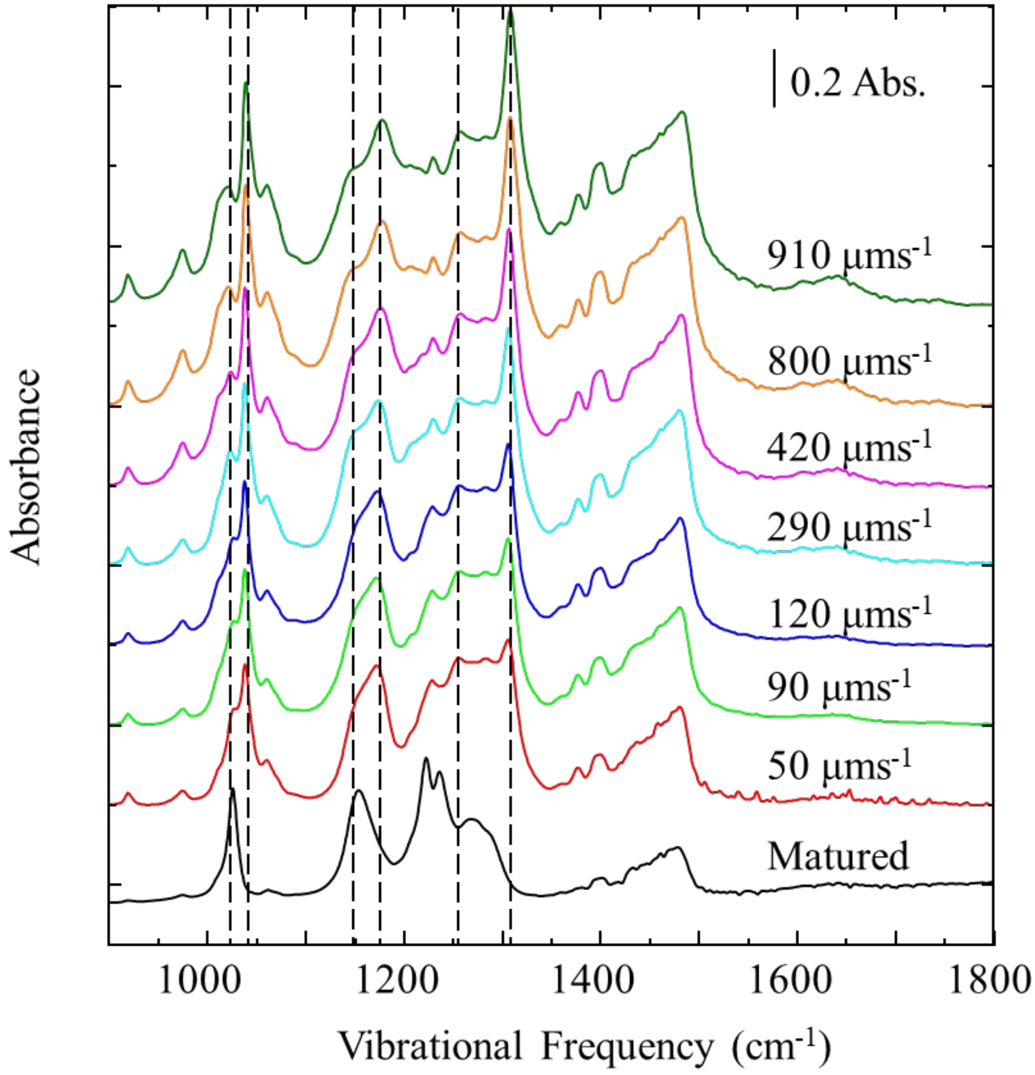

Figure S2: A series of IRRAS spectra showing the changing behavior of a 65/35 [N221H][OTf]/MeCN film as a function of film thickness. The bottom trace represents the matured IL/MeCN film after the substrate rotation is stopped. Above this are the IR traces of IL/MeCN mixture acquired while the substrate is rotating. Films are prepared on a silver substrate using the dynamic wetting technique. Spectra are representative of n≥3 trials.

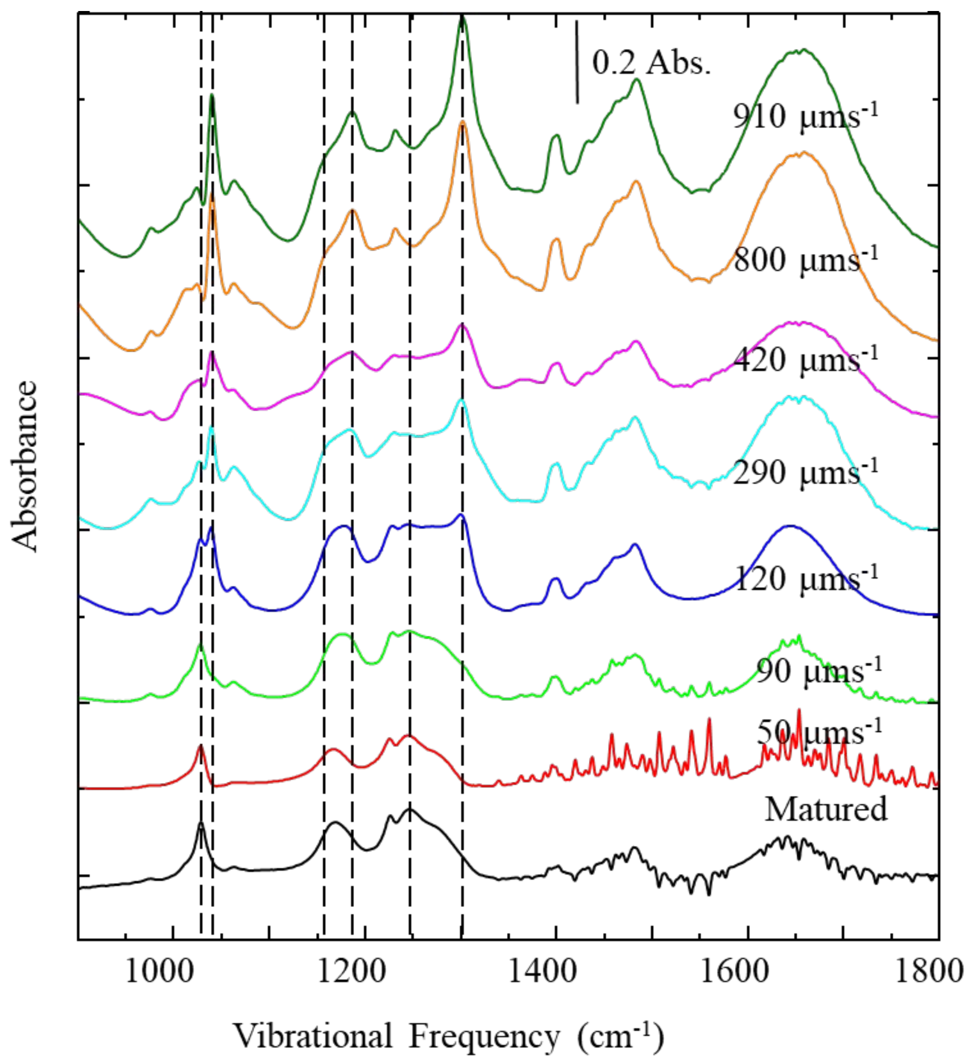

Figure S3: A series of IRRAS spectra showing the changing behavior of a 50/50 [N221H][OTf]/Water film as a function of film thickness. The bottom trace represents the matured IL/Water film after the substrate rotation is stopped. Above this are the IR traces of IL/Water mixture acquired while the substrate is rotating. Films are prepared on a silver substrate using the dynamic wetting technique. Spectra are representative of n≥3 trials.

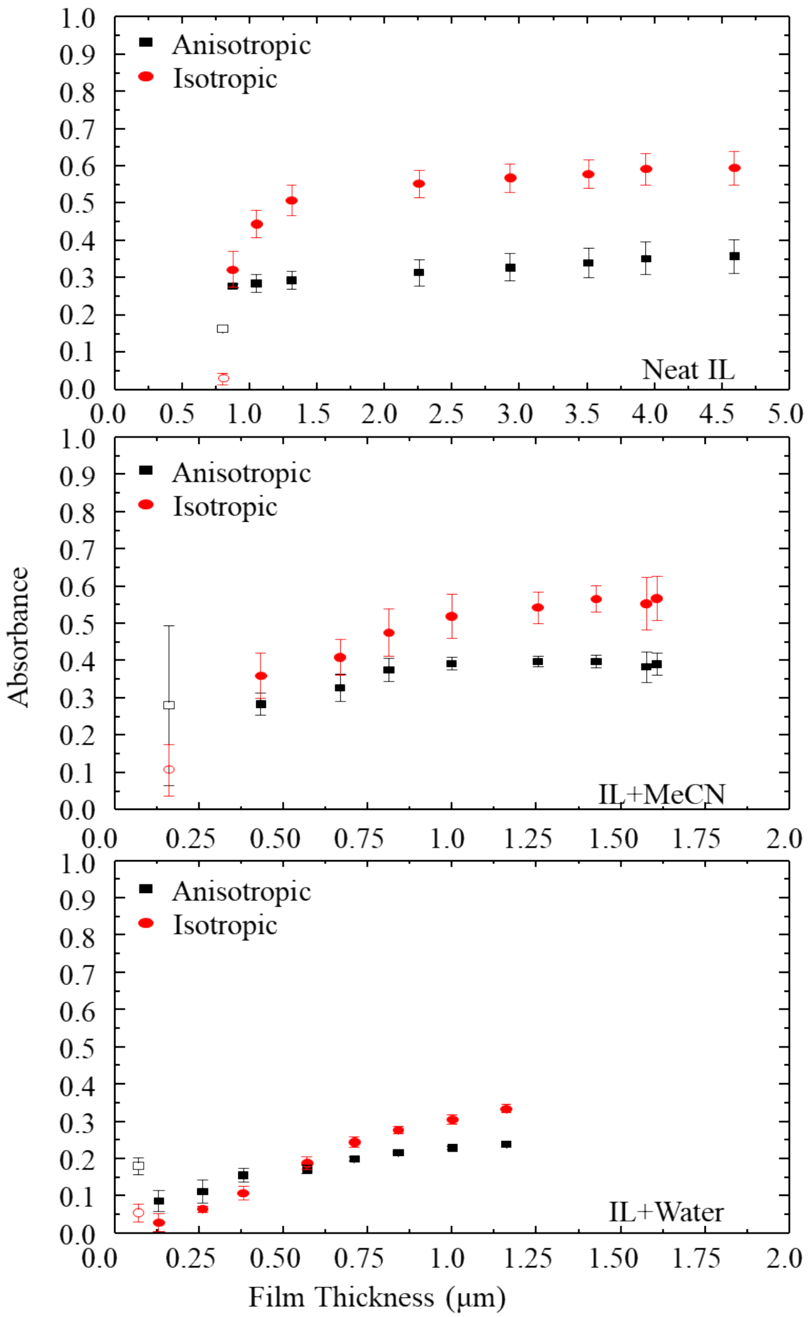

Figure S4: Plots identifying SO_3 symmetric stretch absorbance in the isotropic (1038 cm^{-1}) and anisotropic (1027 cm^{-1}) environments within the liquid film. Data are plotted as a function of film thickness as taken from IRRAS spectra acquired on [BMIM][OTf] (top) and solutions of [BMIM][OTf] and acetonitrile (middle) and [BMIM][OTf] and water (bottom). Films are prepared on a silver substrate using the dynamic wetting technique. Open symbols represent data acquired on the matured film, while filled symbols represent data acquired on the rotating film. Data points are representative of n≥3 trials. Error bars represent the standard deviation in the measurement.

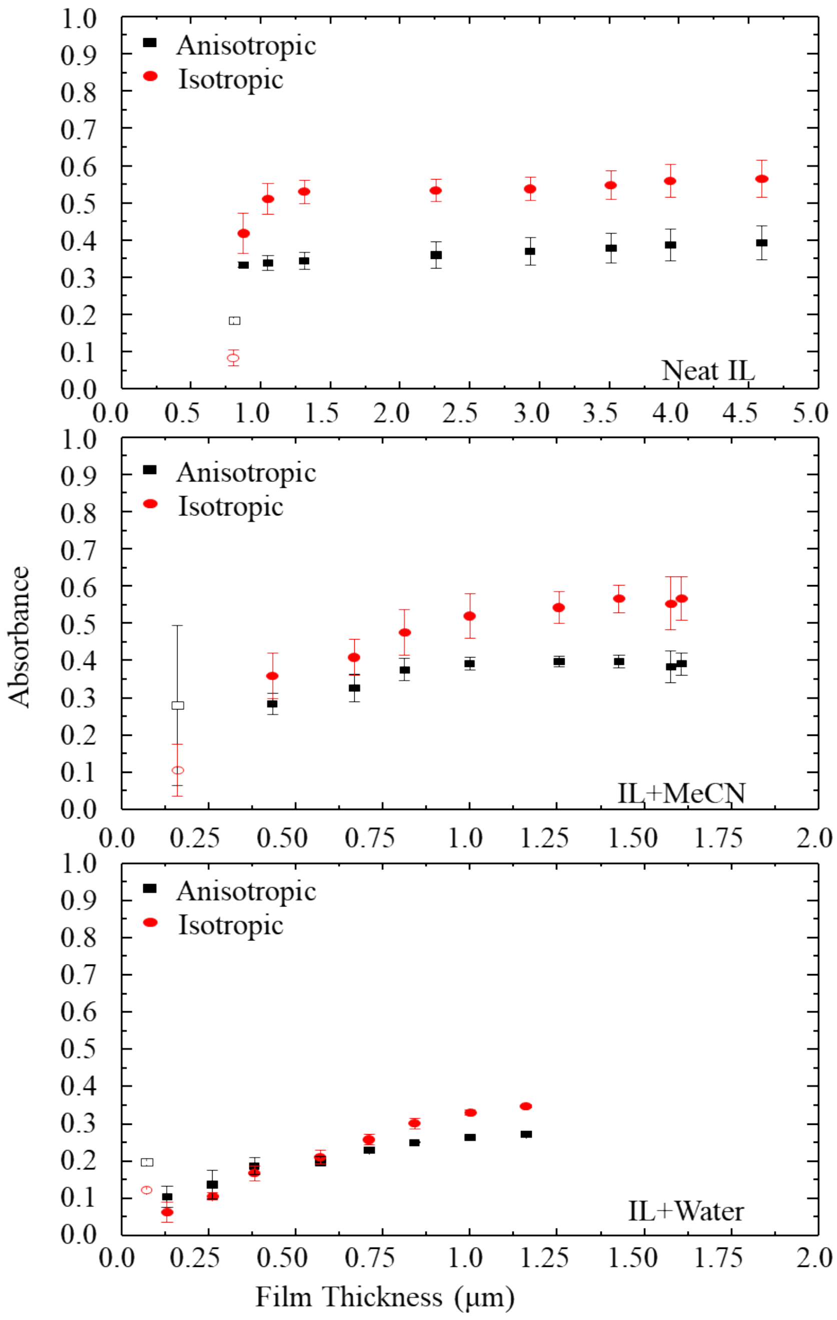

Figure S5: Plots identifying CF_3 asymmetric stretch absorbance in the isotropic (1180 cm^{-1}) and anisotropic (1150 cm^{-1}) environments within the liquid film. Data are plotted as a function of film thickness as taken from IRRAS spectra acquired on [BMIM][OTf] (top) and solutions of [BMIM][OTf] and acetonitrile (middle) and [BMIM][OTf] and water (bottom). Films are prepared on a silver substrate using the dynamic wetting technique. Open symbols represent data acquired on the matured film, while filled symbols represent data acquired on the rotating film. Data points are representative of n≥3 trials.

Neat [BMIM][OTf]						
Film Thickness (μm)	Isotropic SO_3 Symmetric Stretch Absorbance	Anisotropic SO_3 Symmetric Stretch Absorbance	Isotropic SO_3 Asymmetric Stretch Absorbance	Anisotropic SO_3 Asymmetric Stretch Absorbance	Isotropic CF_3 Asymmetric Stretch Absorbance	Anisotropic CF_3 Asymmetric Stretch Absorbance
0.87±0.03	0.32±0.05	0.279±0.006	0.36±0.07	0.307±0.003	0.42±0.05	0.334±0.007
1.05±0.07	0.44±0.04	0.29±0.02	0.54±0.04	0.310±0.02	0.51±0.04	0.34±0.02
1.31±0.08	0.51±0.04	0.29±0.02	0.65±0.05	0.33±0.03	0.53±0.03	0.34±0.02
2.3±0.1	0.55±0.04	0.31±0.03	0.77±0.05	0.35±0.04	0.53±0.03	0.36±0.04
2.9±0.1	0.57±0.04	0.33±0.04	0.81±0.06	0.36±0.03	0.54±0.03	0.37±0.04
3.5±0.2	0.58±0.04	0.34±0.04	0.82±0.06	0.37±0.03	0.55±0.04	0.38±0.04
3.93±0.07	0.60±0.04	0.35±0.04	0.83±0.06	0.37±0.04	0.56±0.04	0.39±0.04
4.6±0.4	0.60±0.07	0.36±0.04	0.84±0.07	0.37±0.04	0.56±0.05	0.39±0.05

Figure S6: Tabulated data of peak absorbance of the isotropic and anisotropic SO_3 symmetric stretch, SO_3 asymmetric stretch, and CF_3 asymmetric stretch for [BMIM][OTf]. Data represent replicate measurements where $n \geq 3$. Error represents standard deviations in the measurement.

60/40 [BMIM][OTf]/MeCN						
Film Thickness (μm)	Isotropic SO_3 Symmetric Stretch Absorbance	Anisotropic SO_3 Symmetric Stretch Absorbance	Isotropic SO_3 Asymmetric Stretch Absorbance	Anisotropic SO_3 Asymmetric Stretch Absorbance	Isotropic CF_3 Asymmetric Stretch Absorbance	Anisotropic CF_3 Asymmetric Stretch Absorbance
0.43±0.09	0.32±0.07	0.25±0.05	0.4±0.1	0.28±.0.05	0.36±0.06	0.28±0.03
0.67±0.08	0.38±0.05	0.28±0.05	0.51±0.08	0.33±0.04	0.41±0.05	0.33±0.04
0.8±0.1	0.45±0.06	0.32±0.03	0.63±0.08	0.37±0.02	0.48±0.06	0.38±0.03
1.0±0.1	0.48±0.06	0.34±0.02	0.67±0.08	0.38±0.01	0.52±0.06	0.39±0.02
1.3±0.1	0.52±0.06	0.34±0.01	0.72±0.08	0.38±0.02	0.54±0.04	0.40±0.01
1.4±0.2	0.54±0.05	0.34±0.02	0.77±0.08	0.39±0.02	0.57±0.04	0.40±0.02
1.57±0.08	0.55±0.07	0.34±0.04	0.8±0.1	0.37±0.04	0.55±0.07	0.38±0.04
1.6±0.1	0.56±0.07	0.34±0.03	0.8±0.1	0.38±0.03	0.57±0.06	0.39±0.03

Figure S7: Tabulated data of peak absorbance of the isotropic and anisotropic SO_3 symmetric stretch, SO_3 asymmetric stretch, and CF_3 asymmetric stretch for 60/40 [BMIM][OTf]/MeCN. Data represent replicate measurements where $n \geq 3$. Error represents standard deviations in the measurement.

50/50 [BMIM][OTf]/Water						
Film Thickness (μm)	Isotropic SO_3 Symmetric Stretch Absorbance	Anisotropic SO_3 Symmetric Stretch Absorbance	Isotropic SO_3 Asymmetric Stretch Absorbance	Anisotropic SO_3 Asymmetric Stretch Absorbance	Isotropic CF_3 Asymmetric Stretch Absorbance	Anisotropic CF_3 Asymmetric Stretch Absorbance
0.13±0.03	0.03±0.02	0.09±0.03	0.03±0.03	0.12±0.02	0.06±0.02	0.11±0.03
0.26±0.02	0.065±0.008	0.11±0.03	0.078±0.01	0.15±0.04	0.11±0.01	0.14±0.04
0.38±0.09	0.11±0.02	0.16±0.02	0.13±0.03	0.19±0.02	0.17±0.02	0.19±0.02
0.54±0.05	0.19±0.02	0.17±0.01	0.23±0.02	0.23±0.01	0.21±0.02	0.20±0.01
0.7±0.1	0.25±0.01	0.199±0.006	0.30±0.03	0.236±0.008	0.26±7.02	0.230±0.008
0.84±0.09	0.28±0.01	0.216±0.002	0.36±0.03	0.257±0.002	0.30±0.01	0.250±0.002
1.0±0.1	0.31±0.01	0.229±0.005	0.38±0.04	0.266±0.002	0.331±0.008	0.264±0.004
1.2±0.2	0.33±0.01	0.240±0.009	0.44±0.02	0.276±0.009	0.348±0.004	0.272±0.009

Figure S8: Tabulated data of peak absorbance of the isotropic and anisotropic SO_3 symmetric stretch, SO_3 asymmetric stretch, and CF_3 asymmetric stretch for 50/50 [BMIM][OTf]/water. Data represent replicate measurements where $n \geq 3$. Error represents standard deviations in the measurement.

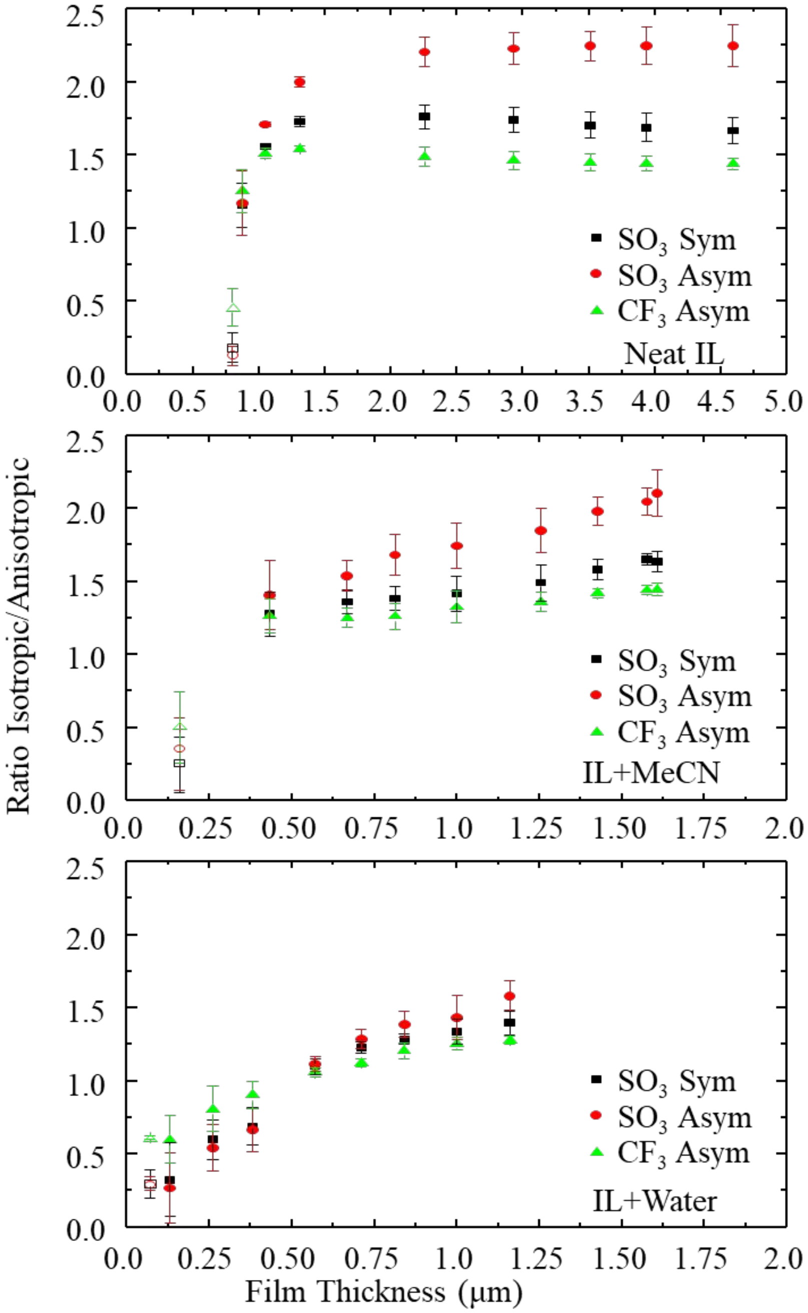

Figure S9: Ratios of the intensity of the peaks corresponding to isotropic modes to the intensity of peaks corresponding to anisotropic modes. Data are plotted as a function of film thickness as taken from IRRAS spectra acquired on neat [BMIM][OTf] (top) and solutions of [BMIM][OTf] and acetonitrile (middle) and [BMIM][OTf] and water (bottom). Peak intensity ratios are show for the SO_3 symmetric stretch (black), SO_3 asymmetric stretch (red), and CF_3 Asymmetric stretch. Films are prepared on a silver substrate using the dynamic wetting technique. Open symbols represent data acquired on the matured film, while filled symbols represent data acquired on the rotating film. Data points are representative of n≥3 trials. Error bars represent the standard deviation in the measurement.

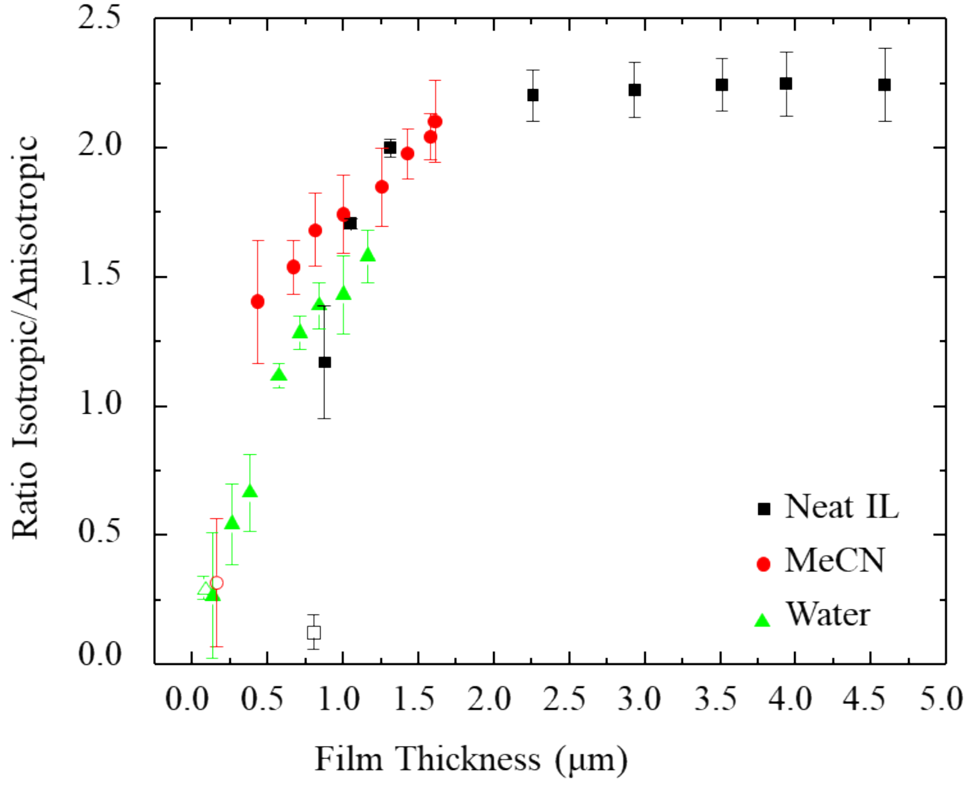

Figure S10: Ratios of the intensity of the peak corresponding to the isotropic SO_3 asymmetric stretch (1300 cm^{-1}) to the intensity of the peak corresponding to the anisotropic SO_3 asymmetric stretch (1250 cm^{-1}). Data are plotted as a function of film thickness as taken from IRRAS spectra acquired on neat [BMIM][OTf] (black) and solutions of [BMIM][OTf] and acetonitrile (red) and [BMIM][OTf] and water (green). Films are prepared on a silver substrate using the dynamic wetting technique. Open symbols represent data acquired on the matured film, while filled symbols represent data acquired on the rotating film. Data points are representative of $n \geq 3$ trials. Error bars represent the standard deviation in the measurement.

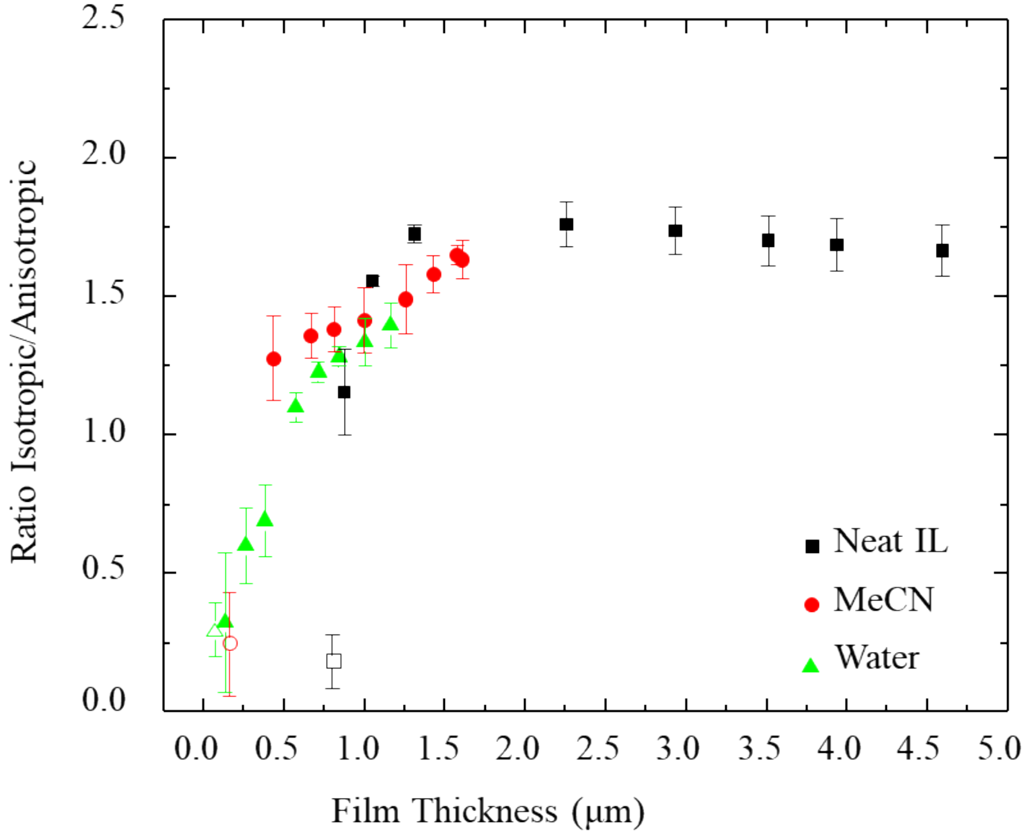

Figure S11: Ratios of the intensity of the peak corresponding to the isotropic SO_3 symmetric stretch (1038 cm^{-1}) to the intensity of the peak corresponding to the anisotropic SO_3 symmetric stretch (1027 cm^{-1}). Data are plotted as a function of film thickness as taken from IRRAS spectra acquired on neat [BMIM][OTf] (black) and solutions of [BMIM][OTf] and acetonitrile (red) and [BMIM][OTf] and water (green). Films are prepared on a silver substrate using the dynamic wetting technique. Open symbols represent data acquired on the matured film, while filled symbols represent data acquired on the rotating film. Data points are representative of $n \geq 3$ trials. Error bars represent the standard deviation in the measurement.

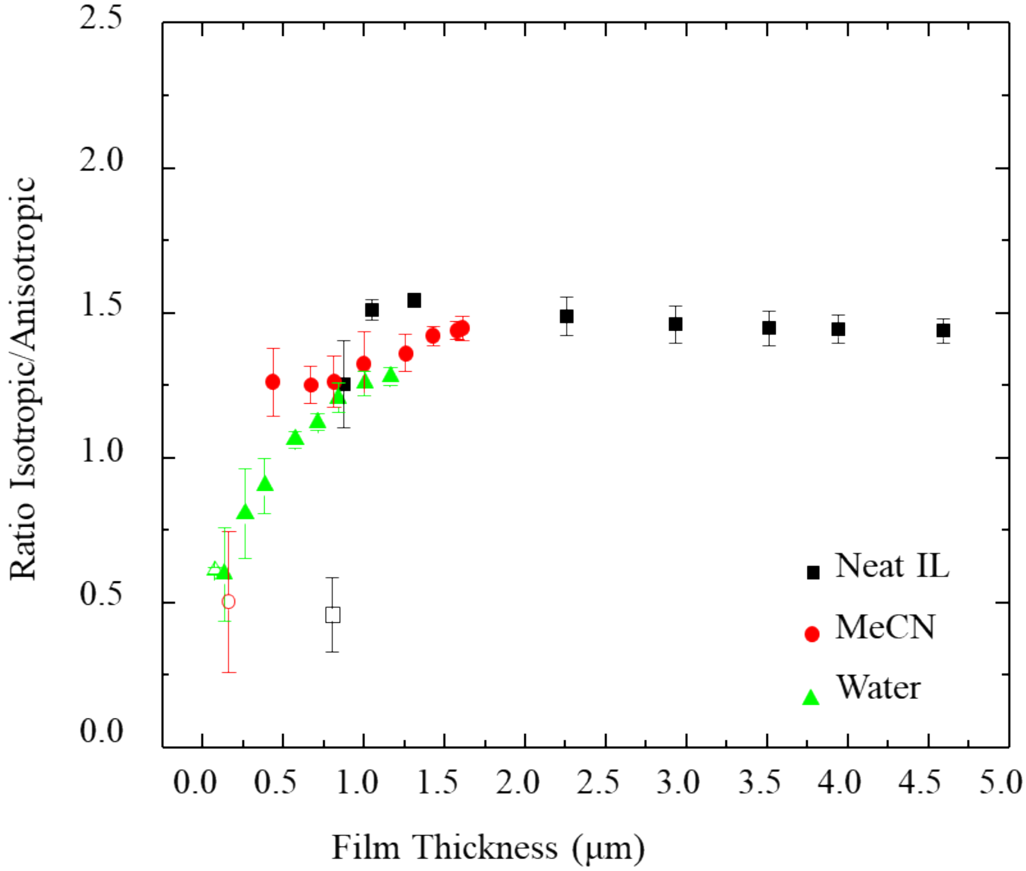

Figure S12: Ratios of the intensity of the peak corresponding to the isotropic CF_3 asymmetric stretch (1180 cm^{-1}) to the intensity of the peak corresponding to the anisotropic CF_3 asymmetric stretch (1150 cm^{-1}). Data are plotted as a function of film thickness as taken from IRRAS spectra acquired on neat [BMIM][OTf] (black) and solutions of [BMIM][OTf] and acetonitrile (red) and [BMIM][OTf] and water (green). Films are prepared on a silver substrate using the dynamic wetting technique. Open symbols represent data acquired on the matured film, while filled symbols represent data acquired on the rotating film. Data points are representative of $n \geq 3$ trials. Error bars represent the standard deviation in the measurement.

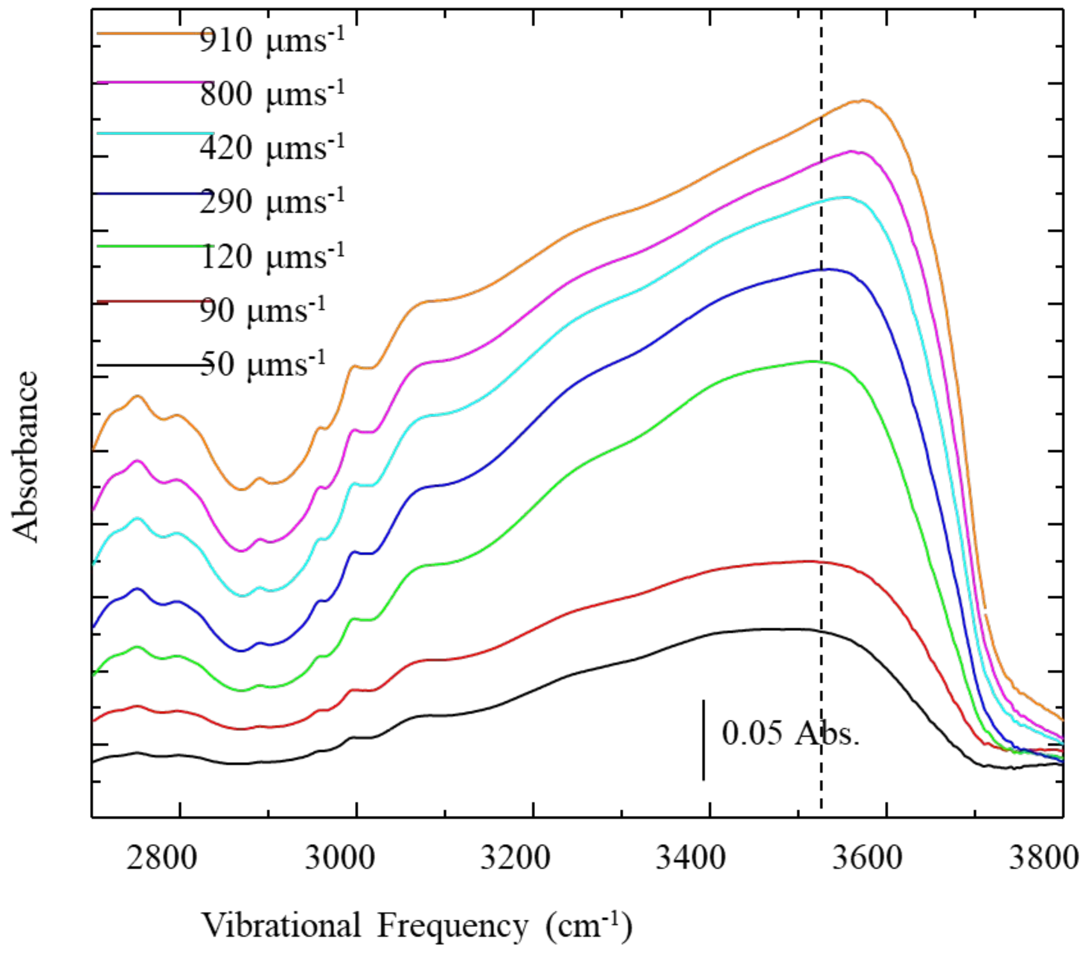

Figure S13: A series of IRRAS spectra showing the changing behavior in the hydrogen bonding region of a 50/50 [N221H][OTf]/Water film as a function of film thickness. The OH stretching mode shifts to higher frequency with film thickness. Films are prepared on a silver substrate using the dynamic wetting technique. Spectra are representative of n≥3 trials.
